# Ancestry inference of 96 population samples using microhaplotypes

**DOI:** 10.1007/s00414-017-1748-6

**Published:** 2017-12-16

**Authors:** Ozlem Bulbul, Andrew J. Pakstis, Usha Soundararajan, Cemal Gurkan, Jane E. Brissenden, Janet M. Roscoe, Baigalmaa Evsanaa, Ariunaa Togtokh, Peristera Paschou, Elena L. Grigorenko, David Gurwitz, Sharon Wootton, Robert Lagace, Joseph Chang, William C. Speed, Kenneth K. Kidd

**Affiliations:** 1 Institute of Forensic Science, Istanbul University, 34098 Istanbul, Turkey; 2 Department of Genetics, Yale University School of Medicine, 333 Cedar Street, New Haven, CT 06520-8005 USA; 3 Turkish Cypriot DNA Laboratory, Committee on Missing Persons in Cyprus Turkish Cypriot Member Office, 99010 (North Cyprus) Nicosia, Turkey; 4 Dr. Fazıl Küçük Faculty of Medicine, Eastern Mediterranean University, 99628 (North Cyprus) Famagusta, Turkey; 5 Department of Medicine, University of Toronto, Toronto, ON M5S Canada; 6 Department of Medicine, The Scarborough Hospital, Toronto, ON M1P 2V5 Canada; 7 Department of Nephrology, Mongolian National University of Medical Sciences, Khoroo 1, Ulaanbataar, Mongolia; 8 Department of Biological Sciences, Purdue University, West Lafayette, IN 47907 USA; 9 Developmental Cognitive Neuroscience, University of Houston, Houston, TX 77204 USA; 10 Laboratory of Translational Sciences of Human Development, St. Petersburg University, St. Petersburg, 199034 Russian Federation; 11 Department of Human Molecular Genetics and Biochemistry, Faculty of Medicine, Tel Aviv University, 69978 Tel Aviv, Israel; 12 Human Identification Group, ThermoFisher Scientific, 180 Oyster Point Blvd, South San Francisco, CA 94080 USA

**Keywords:** Microhaplotype, SNP, Ancestry, Forensics, Massively parallel sequencing (MPS)

## Abstract

Microhaplotypes have become a new type of forensic marker with a great ability to identify and deconvolute mixtures because massively parallel sequencing (MPS) allows the alleles (haplotypes) of the multi-SNP loci to be determined directly for an individual. As originally defined, a microhaplotype locus is a short segment of DNA with two or more SNPs defining three or more haplotypes. The length is short enough, less than about 300 bp, that the read length of current MPS technology can produce a phase-known sequence of each chromosome of an individual. As part of the discovery phase of our studies, data on 130 microhaplotype loci with estimates of haplotype frequency data on 83 populations have been published. To provide a better picture of global allele frequency variation, we have now tested 13 more populations for 65 of the microhaplotype loci from among those with higher levels of inter-population gene frequency variation, including 8 loci not previously published. These loci provide clear distinctions among 6 biogeographic regions and provide some information distinguishing up to 10 clusters of populations.

**Electronic supplementary material:**

The online version of this article (10.1007/s00414-017-1748-6) contains supplementary material, which is available to authorized users.

## Introduction

Microhaplotypes have great ability to identify and deconvolute mixtures because massively parallel sequencing (MPS) allows the alleles (haplotypes) of the multi-SNP loci to be determined directly for an individual [1]. By 2013 [2], our interest in use of haplotypes focused on very short “microhaplotypes.” We have subsequently published on our developing set of microhaplotypes and the criteria for selecting the most useful microhaplotypes for mixture resolution [3–5]. As originally defined, a microhaplotype locus (short form, microhap) is a short segment of DNA with two or more SNPs defining three or more haplotypes at reasonable frequencies in a large part of the world. The loci are designed to be typed with MPS, which can determine, for sequences of up to about 300 bp, the specific combination of SNP alleles on each of the parental chromosomes of an individual. Thus, MPS provides phase-known data, in contrast to conventional Sanger sequencing, at any locus with two or more heterozygous SNPs. Microhaplotype loci have several desirable characteristics including, by definition, multiple alleles. Although most microhaps have fewer alleles than most short tandem repeat polymorphisms (STRPs), microhaps have the advantages over STRPs of very low mutation rates, absence of stutter, and the ability to multiplex large numbers of loci. Sets of microhaplotype loci can be optimized to be useful for individual identification, determining biological relationships, providing information on particular phenotypes, providing information on biogeographic ancestry, or, as noted above, deconvolution of a mixture.

Knowing the haplotype frequency variation around the world is important in determining how useful particular microhaplotypes will be for any one of those five uses in any specific population. Thus, it is important that multiple loci be characterized on as many populations as possible from as many regions of the world as possible. We recently published on 130 microhaplotypes that we have identified and have characterized in 83 populations from around the world [1]. In that paper, we noted the many ways that microhaps can be used but emphasized the value of microhaps for mixture deconvolution. To expand global characterization, we have now collected and analyzed new data on 5667 individuals for 198 SNPs that define 65 microhaplotypes in 13 additional populations, bringing the total from 83 to 96 populations for 65 microhaplotypes. With this broader geographic representation, we now are considering how well microhaps provide information on biogeographic ancestry.

## Materials and methods

### Populations

**Fig. 1:**
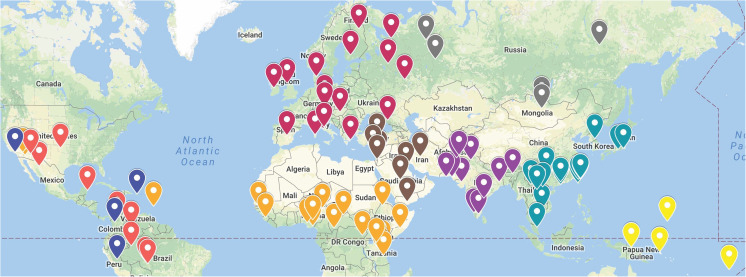
Geographic locations of 96 population samples

Figure 1 shows the geographic locations of the 96 populations (5667 individuals) including 13 new populations. (Two populations that have a cultural-religious basis but no recent single geographic location are omitted from the figure.) The full list of populations is given in Supplemental Table S1. The populations in the table are organized by geographic region. The table also includes the three-character abbreviations used in illustrations, and the unique sample identifier (UID) in the ALFRED database <https://alfred.med.yale.edu> [6, 7] for the description of each sample. The new population samples had only small amounts of DNA available and were chosen, in part, to provide somewhat more uniform sampling of populations around the world. All samples were collected with full informed consent per local law allowing studies such as this.

### Selection of loci

To characterize the additional populations and emphasize ancestry inference, we chose loci with higher ranks by informativeness (*I*_*n*)_ based on the 83 populations already evaluated for all 130 microhaplotypes and additional loci from among those loci subsequently characterized [1]. Eight loci not previously published are noted in Table 1. Availability of TaqMan assays already on hand in the laboratory determined which loci were specifically tested first. The current set of 65 loci involves 198 SNPs and represents an empiric balance of available assays and sufficient DNA. Additional loci may be tested on some of these populations in the future, but the available DNA has been exhausted for several of these “new” populations.

**Table 1:** Definitions of eight new and previously unpublished microhaplotypes

Locus name	Gene region	SNPs included	Build 38 nt positions
mh02KK-105	FER1L5	rs2280355/rs2280356	96700566 96700587
mh03KK-020	CLSTN2	rs4683510/rs12494698	140566273 140566492
mh05KK-122	SLC45A2	rs1010872/rs28777	33958805 33958854
mh05KK-123	SLC45A2	rs28117/rs1423676	33962665 33962772
mh05KK-124	SLC45A2	rs35414/rs3756464	33969523 33969589
mh06KK-030	ATXN1	rs10949381/rs675934/rs607341	16801536 16801552 16801635
mh06KK-031	BAI3 - ADGRB3	rs10455681/rs10455682	69092610 69092768
mh16KK-061	ZCCHC14	rs4559917/rs6540049	87447773 87447822

### Genotyping

All markers were typed using TaqMan assays obtained from Thermo Fisher. The individuals with large amounts of DNA were typed following manufacturer’s protocols with reaction volumes reduced to 3 μl, run in 384-well plates, and read on an AB9700HT using Applied Biosystems’ SDS (sequence detection system) software. To maximize the number of SNPs that could be typed on the small amounts of DNA available, a pre-amplification protocol was employed as described [8].

### Haplotyping

The haplotypes were estimated using phase version 2.1.1. [9, 10] as described previously [1]. This approach provides good allele frequency estimates for these reference populations.

### Statistics

The effective number of alleles, *A*_*e*_, was calculated following Kidd and Speed [5]. Informativeness, *I*_*n*_, was calculated using the formula of Rosenberg et al. [11]. STRUCTURE analyses [12] were done for the full set of 96 populations and 65 loci with 10 independent runs at each *K* value. PCA was calculated with Addinsoft’s XLSTAT 2017.

## Results

Table 1 lists the 8 previously unpublished loci and their definitions using the nomenclature for microhaplotypes we previously proposed [13]. The definitions of all 65 loci, including those previously published, are in ALFRED <alfred.med.yale.edu>. The allele frequencies for all 65 loci, including the 8 new loci, are now in ALFRED for all 96 populations along with the data on the rest of the original 130 microhaps [1]. Data on all these loci can be retrieved using the key word *microhap* on the ALFRED home page or on the drop down *Search* menu.

**Fig. 2:**
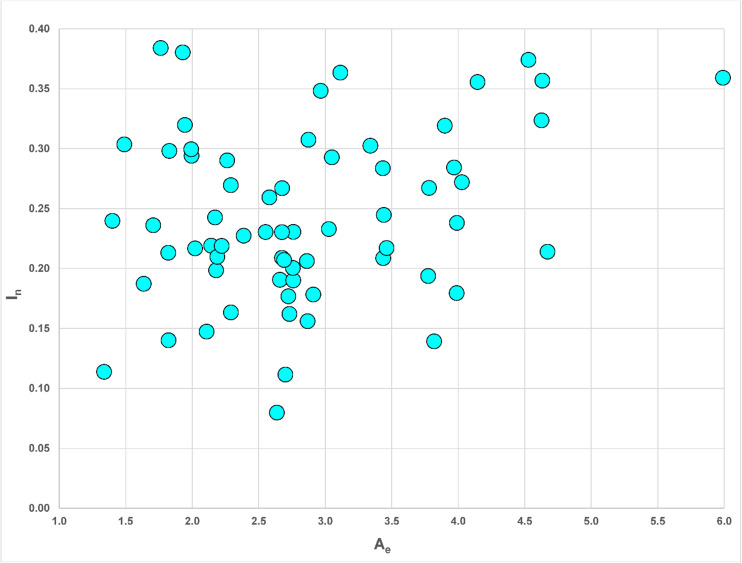
Scatterplot of average effective allele number (*A*_*e*_) by informativeness (*I*_*n*_) for 65 microhaplotypes studied on 96 populations based on the values in Table 2

Table 2 lists all 65 microhaplotypes along with their *A*_*e*_ and *I*_*n*_ values, with the ranks from largest to smallest, using all 96 populations to calculate the statistics. Figure 2 is a scatterplot of the 65 loci by these two statistics. Comparison of the scatterplot in Fig. 2 with that in [1] shows that these 65 loci have proportionately fewer loci with *I*_*n*_ less than 0.1 and *A*_*e*_ less than 2.0.

**Table 2:** The 65 microhaplotype loci and their average effective number of alleles (*A*_*e*_) and Informativeness (*I*_*n*_) values for 96 populations

Locus name		Global avg. *A*_*e*_ 96 pops	*A*_*e*_ rank	*I*_*n*_ 96 pops	*I*_*n*_ rank
mh01KK-001		3.114	20	0.363	4
mh01KK-002		2.676	35	0.267	25
mh01KK-106		2.581	40	0.259	26
mh01KK-117		3.969	10	0.284	20
mh01KK-205		3.819	12	0.139	62
mh02KK-003		1.929	56	0.380	2
mh02KK-004		2.552	41	0.230	34
mh02KK-102		1.636	62	0.187	53
mh02KK-105	New	2.022	52	0.217	40
mh02KK-134		4.528	5	0.374	3
mh02KK-136		3.774	14	0.194	50
mh02KK-201		2.172	49	0.243	28
mh03KK-006		1.821	59	0.213	42
mh03KK-020	New	1.996	53	0.294	17
mh04KK-010		2.723	32	0.177	56
mh04KK-013		3.781	13	0.267	24
mh04KK-015		2.109	51	0.147	60
mh04KK-017		2.675	37	0.209	44
mh05KK-062		2.143	50	0.219	37
mh05KK-122	New	1.993	54	0.299	15
mh05KK-123	New	2.180	48	0.198	49
mh05KK-124	New	2.291	44	0.269	23
mh06KK-030	New	2.660	38	0.191	51
mh06KK-031	New	1.489	63	0.304	13
mh06KK-101		1.707	61	0.236	31
mh08KK-032		2.292	43	0.163	57
mh09KK-034		2.189	47	0.210	43
mh09KK-035		2.637	39	0.080	65
mh09KK-152		2.863	27	0.206	47
mh09KK-153		2.966	23	0.348	8
mh09KK-157		3.441	16	0.245	27
mh10KK-163		4.627	4	0.324	9
mh10KK-169		4.633	3	0.357	6
mh11KK-037		2.222	46	0.219	38
mh11KK-040		2.263	45	0.290	19
mh11KK-091		1.822	58	0.140	61
mh11KK-180		4.028	7	0.272	22
mh11KK-191		3.027	22	0.233	32
mh12KK-046		2.868	26	0.156	59
mh13KK-047		2.387	42	0.227	36
mh13KK-217		3.991	8	0.238	30
mh13KK-218		5.991	1	0.359	5
mh13KK-225		3.436	17	0.209	45
mh14KK-048		2.760	29	0.190	52
mh14KK-101		1.764	60	0.384	1
mh15KK-066		2.757	30	0.200	48
mh15KK-067		2.761	28	0.230	33
mh16KK-061	New	2.691	34	0.207	46
mh16KK-096		1.401	64	0.240	29
mh16KK-255		3.434	18	0.284	21
mh16KK-302		2.875	25	0.307	12
mh17KK-052		2.701	33	0.111	64
mh17KK-105		1.338	65	0.114	63
mh17KK-272		2.911	24	0.178	55
mh18KK-285		2.675	36	0.230	35
mh18KK-293		3.340	19	0.302	14
mh19KK-299		3.899	11	0.319	11
mh19KK-301		1.828	57	0.298	16
mh20KK-058		2.732	31	0.162	58
mh20KK-307		3.462	15	0.217	39
mh21KK-315		3.989	9	0.180	54
mh21KK-316		3.050	21	0.293	18
mh21KK-320		4.673	2	0.214	41
mh21KK-324		4.146	6	0.356	7
mh22KK-069		1.946	55	0.320	10
Average		2.849		0.243	
Variance		0.881463145		0.005007238

**Fig. 3:**
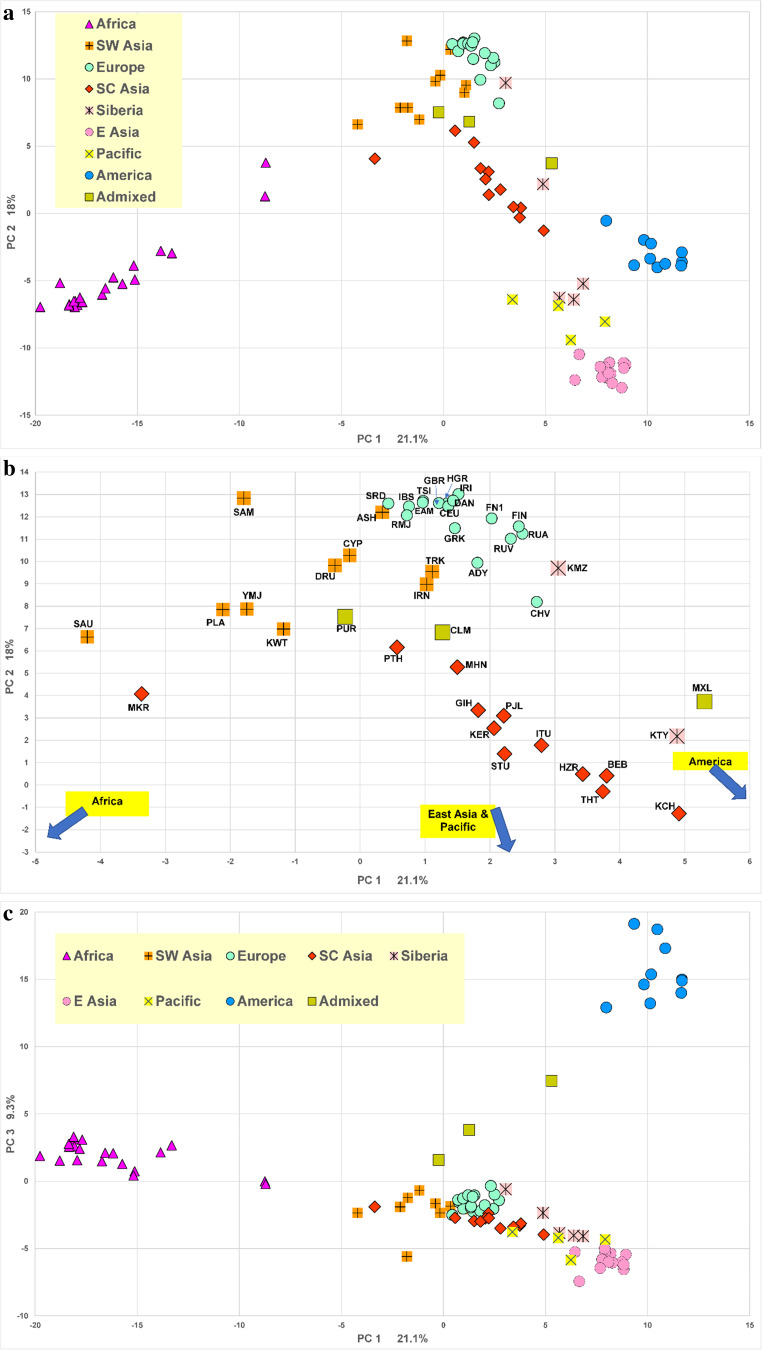
Principal components analysis scatter plots for the 96 populations analyzed using the 65 microhap dataset. Fig. 3a plots PC1 × PC2. Fig. 3b shows enlarged view from Fig. 3a near Southwest Asian, European, and South Central Asian populations. Fig. 3c plots PC1 × PC3

Figure 3 presents scatterplots from the principal components analysis (PCA) of the 96 populations based on their allele frequencies at the 65 loci. The first two PCs account for 39% of the variation (Fig. 3a); the major continental regions––Africa, Southwest Asia, Europe, South Central Asia, East Asia, Americas, and Pacific—clearly separate from one another. Figure 3b shows an enlarged view of the tighter cluster of populations with labels for the individual populations following the labels in Supplemental Table S1. The third PC accounts for an additional 9.28% (Fig. 3c) and separates the Native American populations more distinctly from the East Asian populations. The fourth PC (not shown) accounts for only an additional 4% of the variation and moves the Pacific Island populations away from East Asia.

**Fig. 4:**
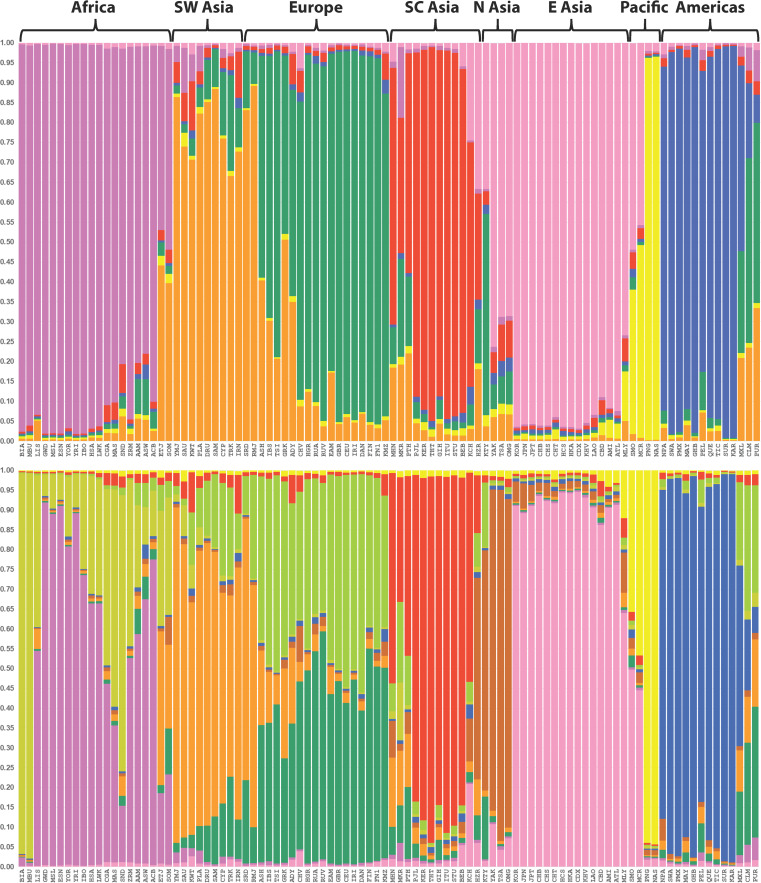
Estimated cluster membership values in STRUCTURE analyses for 96 populations at *K* = 7 and *K* = 10 (highest likelihood run results). See Supplemental Table S1 for population details

Figure 4 shows population averages for STRUCTURE analyses at *K* = 7 and *K* = 10. At *K* = 7, except for small amounts of “noise,” the majority of Africans are assigned to a single cluster. The majority of East Asians are assigned to a single cluster, and the majority of the Native Americans are assigned to a single cluster. Three of the “Americas” populations from the 1000 Genomes project [14] are highly admixed in these analyses and are labeled as such. At *K* = 10, the populations farthest North (Eastern Siberia, Mongolia) in East Asia cluster together apart from other East Asians while the Sub-Saharan African populations subdivide into distinctive patterns for West Africa compared to Central and East African populations.

Comparison of the STRUCTURE results at *K* = 10 with Fig. 3b shows, by the two distinct statistics, that information on the finer relationships is present in the dataset.

## Discussion

Forensic questions that can be addressed by microhaps include resolution of mixtures, identifying relatives, inferring ancestral origins, estimating phenotype, and individualization. In this study, we have emphasized ancestral origins, but we note that this set of 65 microhaplotypes spread across 21 human autosomes can be very useful for mixture deconvolution, familial inference, and individualization as well. Microhaplotypes can incorporate SNPs useful for estimating aspects of phenotype, but we have not considered that type of information. Our analyses of these 65 loci have focused on biogeographic ancestry. In future papers, we will present analyses of random match probabilities (RMP) and familial inference for sets of microhaps. We note that we have estimated the RMP for the reference CEU population sample for these 65 loci; the estimated RMP is between 10^−55^ and 10^−56^.

These 65 loci have different characteristics as measured by *A*_*e*_ and *I*_*n*_. Those differences are shown in Fig. 2. We note that the two microhaps with the highest *I*_*n*_ values (mh14KK-101 and mh02KK-003) are among the loci with the lowest *A*_*e*_ values (ranks 60 and 56, respectively, Table 2). Examination of the haplotype frequencies shows that the global patterns are noticeably different and have large regions of the world with one haplotype at greater than 80% frequencies, albeit different haplotypes in different regions (Supplemental Fig. 2). The other six loci with *I*_*n*_ > 0.35 show a range of *A*_*e*_ values from nearly three to nearly six. Basically, these loci have multiple alleles everywhere but very different allele frequencies. We also note that some of the loci have low ranks for both measures.

The PCA in Fig. 3 shows that this set of microhaps distinguish among populations from some of the different biogeographic regions. Africa and the Americas are very clearly distinct. The Eastern and Northern of the Eurasian populations also fall into a loose cluster clearly distinct from European populations. What is interesting is how relatively close the European populations cluster in this global set of populations. On the other hand, this is not surprising because Europe is a geographically small area. Figure 3b makes clear how geographic boundaries do not reflect the genetic clustering of populations. The Komi from NW Siberia are clearly “European”; the Khanty from W Siberia are intermediate between European and the Northern Asian populations from Mongolia and Eastern Siberia.

The global dispersion is reflected in the STRUCTURE results in Fig. 4. At *K*=7, there are quite distinct biogeographic regions with intermediate populations showing partial assignment to flanking clusters. Europe and Southwest Asia are the exception in that a Southwest to Northeast cline of two clusters is seen. This is a pattern that has been seen with many sets of ancestry markers [e.g., 15, 16].

Our previous paper studying 130 loci on 83 populations [1] had 28 loci with *A*_*e*_ > 3.0 giving a very high probability of identifying and resolving a mixture of two individuals; the current dataset has 22 loci in this range. By definition, these 22 loci have multiple alleles and relatively low frequencies for each of the alleles. These same attributes make these good for identifying relatives and make the likelihood of two unrelated individuals having the same multi-locus genotype vanishingly small.

Three of the new microhaps are in the region of SLC45A2. The coding SNP at SLC45A2 that is associated with skin color, rs16891982, is located on chromosome 5 at nt 33,951,588 (build 38) and shows a different global pattern of variation from even the closest of these three microhaps which is 17 kb away. The three microhaps also differ in their global patterns (Supplemental Fig. 1). At this stage, we are not choosing which is better since the different microhaps may differ in appropriateness for different purposes. The global variation is not identical, with *I*_*n*_ values from a high of 0.299 (rank 15) for mh05KK-122 to 0.198 (rank 49) for mh05KK-123. In the context of 65 loci with multiple alleles, including all three will have little effect other than strengthening whatever pattern in STRUCTURE analyses is favored by that chromosomal region––Europe and SW Asia are distinct from East Asia.

## Conclusions

The addition of 13 populations and emphasis on microhaplotype loci with higher *I*_*n*_ values on average, compared to the 130 microhaps in [1], has shown that these 65 loci constitute a significant panel for ancestry inference. These results provide additional material for selecting panels of microhaplotypes optimized for different purposes.

That many of the loci also have high *A*_*e*_ values argues that most of these loci have value for mixture deconvolution. The overall results support our previous findings [1] that many microhaplotypes have use for both ancestry inference and mixture deconvolution. The markers with *A*_*e*_ > 3.0 are particularly good at mixture deconvolution, and the same logic indicates they will be very useful for familial relationships and individualization.

As we have been able to identify and characterize more microhaplotypes as part of our discovery phase, it has become possible to begin the laboratory studies to determine which loci will be the most robust with actual MPS typing. Our objective has been to identify and characterize a large number of loci with useful statistical characteristics such that molecular issues that may exclude some loci from MPS multiplexes will leave sufficient loci. These studies provide the necessary estimates of reference population allele frequencies. When actual multiplex kits are available, the identification of microhaplotype genotypes in individuals using MPS promises to be an important adjunct to forensic casework.

## Electronic supplementary material

ESM 1 (PDF 665 kb)
