# Supplementary material for: Ancestry inference of 96 population samples using microhaplotypes
Source: Int J Legal Med. 2017 Dec 16;132(3):703–11. doi: 10.1007/s00414-017-1748-6 (PMC5920014; doi:10.1007/s00414-017-1748-6)

SUPPLEMENTARY MATERIAL

Submitted to International Journal of Legal Medicine
for manuscript titled:

Ancestry inference of 96 population samples using microhaplotypes

Ozlem Bulbul[1], Andrew J. Pakstis[2], Usha Soundararajan[2], Cemal Gurkan[3,4], Jane E. Brissenden[5], Janet M. Roscoe[5,6], Baigalmaa Evsanaa[7], Ariunaa Togtokh[7], Peristera Paschou[8], Elena L. Grigorenko[9,10], David Gurwitz[11], Sharon Wootton[12], Robert Lagace[12], Joseph Chang[12], William C. Speed[2], Kenneth K. Kidd[2]

1 Institute of Forensic Science, Istanbul University, Istanbul, 34098, Turkey

2 Department of Genetics, Yale University School of Medicine, New Haven, CT 06520 USA

3 Turkish Cypriot DNA Laboratory, Committee on Missing Persons in Cyprus Turkish Cypriot Member Office, Nicosia 99010 (North Cyprus), Turkey

4 Dr. Fazıl Küçük Faculty of Medicine, Eastern Mediterranean University, Famagusta, 99628 (North Cyprus), Turkey

5 Department of Medicine, University of Toronto, Toronto, ON M5S, Canada

6 Department of Medicine, The Scarborough Hospital, Toronto, ON M1P 2V5, Canada

7 Department of Nephrology, Mongolian National University of Medical Sciences, Khoroo 1, Ulaanbataar, Mongolia

8 Department of Biological Sciences, Purdue University, West Lafayette, 47907, Indiana, USA

9 Developmental Cognitive Neuroscience, University of Houston, Houston, TX 77204 USA

10 Institute of Molecular Medicine, University of North Texas Health Science Center, 3500 Camp Bowie Blvd. Fort Worth, TX 76107, USA

11 Department of Human Molecular Genetics and Biochemistry, Faculty of Medicine, Tel Aviv University, Tel Aviv 69978, Israel

12 Human Identification Group, ThermoFisher Scientific, 180 Oyster Point Blvd, South San Francisco, CA, 94080, USA

Corresponding author:
Kenneth K. Kidd, Ph.D.
Professor Emeritus
Senior Research Scientist
Department of Genetics
Yale University School of Medicine
333 Cedar Street
New Haven, CT 06520-8005 USA
Telephone: 203-785-2654
Email: Kenneth.Kidd@yale.edu

Supplemental Table S1. The 96 population samples analyzed. Data on these populations were collected in the Kidd Lab except for the data on populations from the 1000 Genomes Project (1KG). Populations in Figure 4 appear in the same order as in this table.

Sample Size N	Abbrev		ALFRED Sample Population UID	Population sample description and World Region
				**Africa**
68	BIA		SA000005F	Biaka
38	MBU		SA000006F	Mbuti
8	LIS		SA002770Q	Lisongo
113	GWD	1KG	SA004243N	Gambians, Western Division,The Gambia
85	MSL	1KG	SA004244O	Mende, Sierra Leone
99	ESN	1KG	SA004248S	Esan, Nigeria
77	YOR		SA000036J	Yoruba, Benin City, Nigeria
108	YRI	1KG	SA004048Q	Yoruba, Ibadan, Nigeria
47	IBO		SA000099S	Ibo, Nigeria
39	HSA		SA000100B	Hausa, Nigeria
99	LWK	1KG	SA003388W	Luhya, Webuye, Kenya
43	CGA		SA000487T	Chagga, Tanzania
20	MAS		SA000854R	Masai, Tanzania
40	SND		SA001773S	Sandawe, Tanzana
38	ZRM		SA002586V	Zaramo, Tanzania
87	AAM		SA000101C	African Americans
61	ASW	1KG	SA004047P	African Americans, SW USA
96	ACB	1KG	SA004242M	AfroCaribbeans, Barbados
31	ETJ		SA000015G	Ethiopian Jews
5	SOM *		SA002138O	Somali, collected in Pakistan
				**Southwest Asia**
40	YMJ		SA000016H	Yemenite Jews
51	SAU *		SA004393T	Saudi
15	KWT		SA002765U	Kuwaiti
58	PLA *		SA002766V	Palestinian Arabs
125	DRU		SA000047L	Druze, Israel
39	SAM		SA000098R	Samaritans, Israel
36	CYP *		SA004333N	Turkish Cypriots
77	TRK *		SA004310I	Turkish, Istanbul, Turkey
35	IRN *		SA004309Q	Iranians
				**Europe**
35	SRD		SA002768X	Sardinians
26	RMJ		SA000096P	Roman Jews, Italy
77	ASH		SA000490N	Ashkenazi Jews
107	IBS	1KG	SA004108N	Iberians
107	TSI	1KG	SA004057Q	Toscani, Italy
50	GRK *		SA002767W	Greeks,Thesaloniki, Greece
54	ADY		SA000017I	Adygei

42	CHV		SA000491O	Chuvash
142	HGR		SA002023H	Hungarians
30	RUA		SA001530J	Russians, Archangelsk
47	RUV		SA000019K	Russians, Vologda
85	EAM		SA000020C	European Americans
91	GBR	1KG	SA004050J	British from England and Scotland
99	CEU	1KG	SA004250L	Utah Residents (CEPH), N&W Europe ancestry
113	IRI		SA000057M	Irish
51	DAN		SA000007H	Danes
34	FIN		SA000018J	Finns
99	FN1	1KG	SA004049R	Finns, Finland
46	KMZ		SA000489V	Komi Zyriane
				**South Central Asia**
22	MHN *		SA002139P	Mohanna, Pakistan
6	MKR *		SA002137N	Negroid Makrani, Pakistan
14	PTH *		SA002873U	Pathans, Pakistan
96	PJL	1KG	SA004240K	Punjabi, Lahore, Pakistan
30	KER		SA001854S	Keralites, India; collected in Connecticut USA
13	THT		SA000077O	Thoti, India
103	GIH	1KG	SA004246Q	Gujarati (India), Houston, Texas
102	ITU	1KG	SA004247R	Telugu (India), collected in United Kingdom
102	STU	1KG	SA004241L	Tamil (Sri Lanka), collected in United Kingdom
86	BEB	1KG	SA004239S	Bengali, Bangladesh
16	KCH		SA000040E	Kachari, Assam state, India
25	HZR *		SA002140H	Hazara, Pakistan
				**North Asia**
45	KTY		SA000488U	Khanty
50	YAK		SA000011C	Yakut
36	TSA *		SA004036N	Tsaatan
44	OMG *		SA004035N	Outer Mongolians
				**East Asia**
54	KOR		SA000936S	Koreans
47	JPN		SA000010B	Japanese
104	JPT	1KG	SA004060K	Japanese, Tokyo, Japan
103	CHB	1KG	SA004058R	Han Chinese in Bejing, China
58	CHS		SA000009J	Chinese, San Francisco, USA
47	CHT		SA000001B	Chinese, Taiwan
105	HCS	1KG	SA004059S	Southern Han Chinese
41	HKA		SA000003D	Hakka
93	CDX	1KG	SA004238R	Chinese Dai in Xishuangbanna, China
99	KHV	1KG	SA004249T	Kinh (Vietnamese), Ho Chi Minh City, Vietnam
118	LAO		SA001853R	Laotians
24	CBD		SA000022E	Cambodians
38	AMI		SA000002C	Ami, Taiwan
40	ATL		SA000021D	Atayal, Taiwan
11	MLY		SA000097Q	Malaysians

				**Pacific**
9	SMO		SA000072J	Samoans
34	MCR		SA000063J	Micronesians
22	PNG		SA000084M	Papuans, New Guinea
23	NAS		SA000012D	Nasioi, Bougainville, Solomon Islands
				**Americas**
54	NPA		SA000023F	Plains AmerIndians
51	SWA		SA000025H	Southwest AmerIndians
98	PMX		SA000026I	Pima, northern Mexico
49	MAY		SA000013E	Maya, Yucatan, Mexico
12	GHB		SA000055K	Guihiba speakers, Colombia
85	PEL	1KG	SA004245P	Peruvians, Lima, Peru
22	QUE		SA000069P	Quechua, Peru
65	TIC		SA000027J	Ticuna, Amazon region, Brazil
42	SUR		SA000014F	Rondonian Surui, Amazon region, Brazil
54	KAR		SA000028K	Karitiana, Amazon region, Brazil
				**Admixed groups, Americas**
64	MXL	1KG	SA004110G	Mexican Americans, LosAngeles
94	CLM	1KG	SA004109O	Colombians from Medellin
104	PUR	1KG	SA004111H	PuertoRicans

Notes:
The 13 newest populations included in microhaplotype analyses are noted by an asterisk (*) next to the population abbreviation.

Supplemental Figure S1. Allele frequency bar graphs at SLC45A2 for four loci

rs16891982

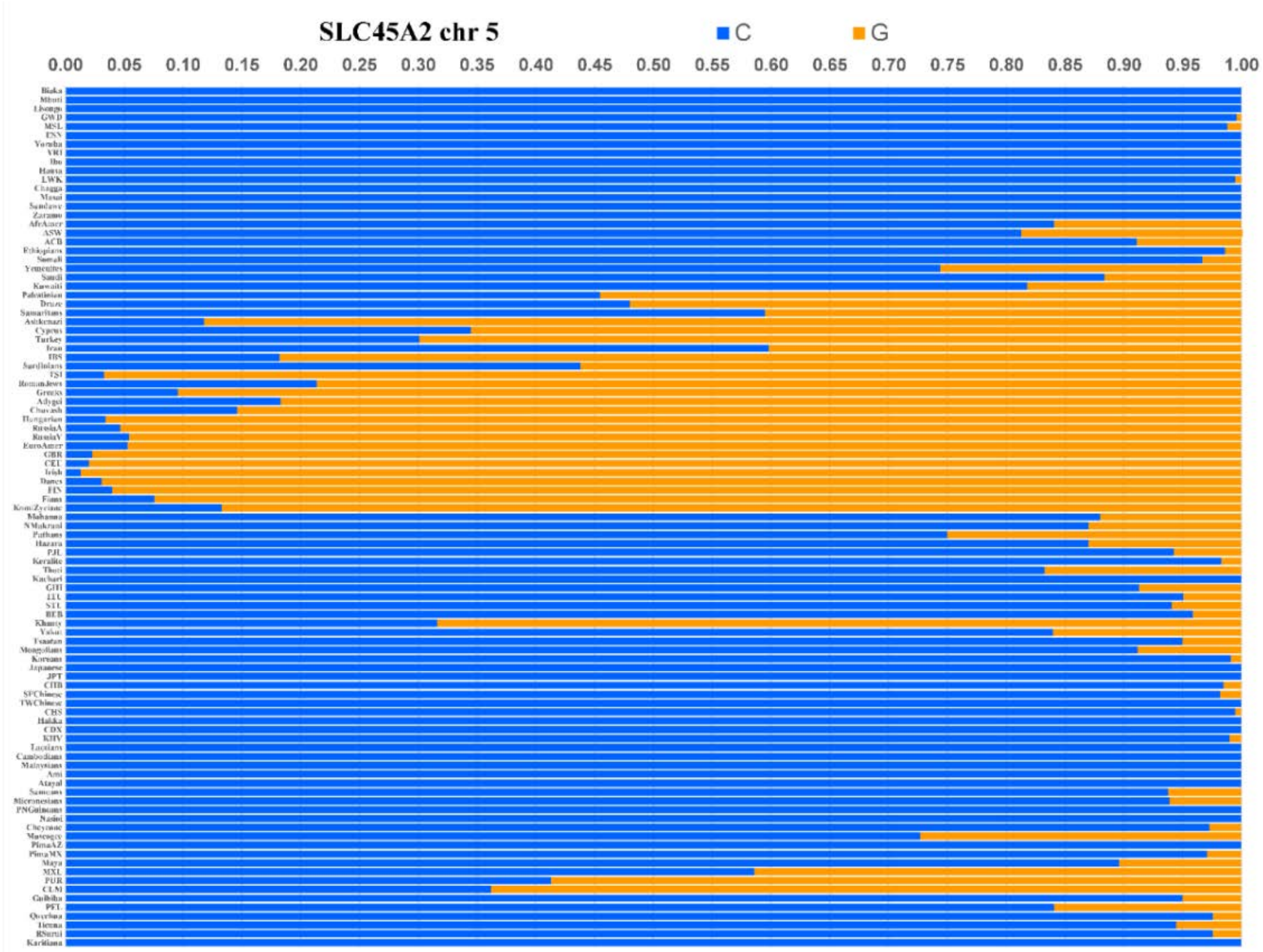

Microhaplotype MH05KK-122

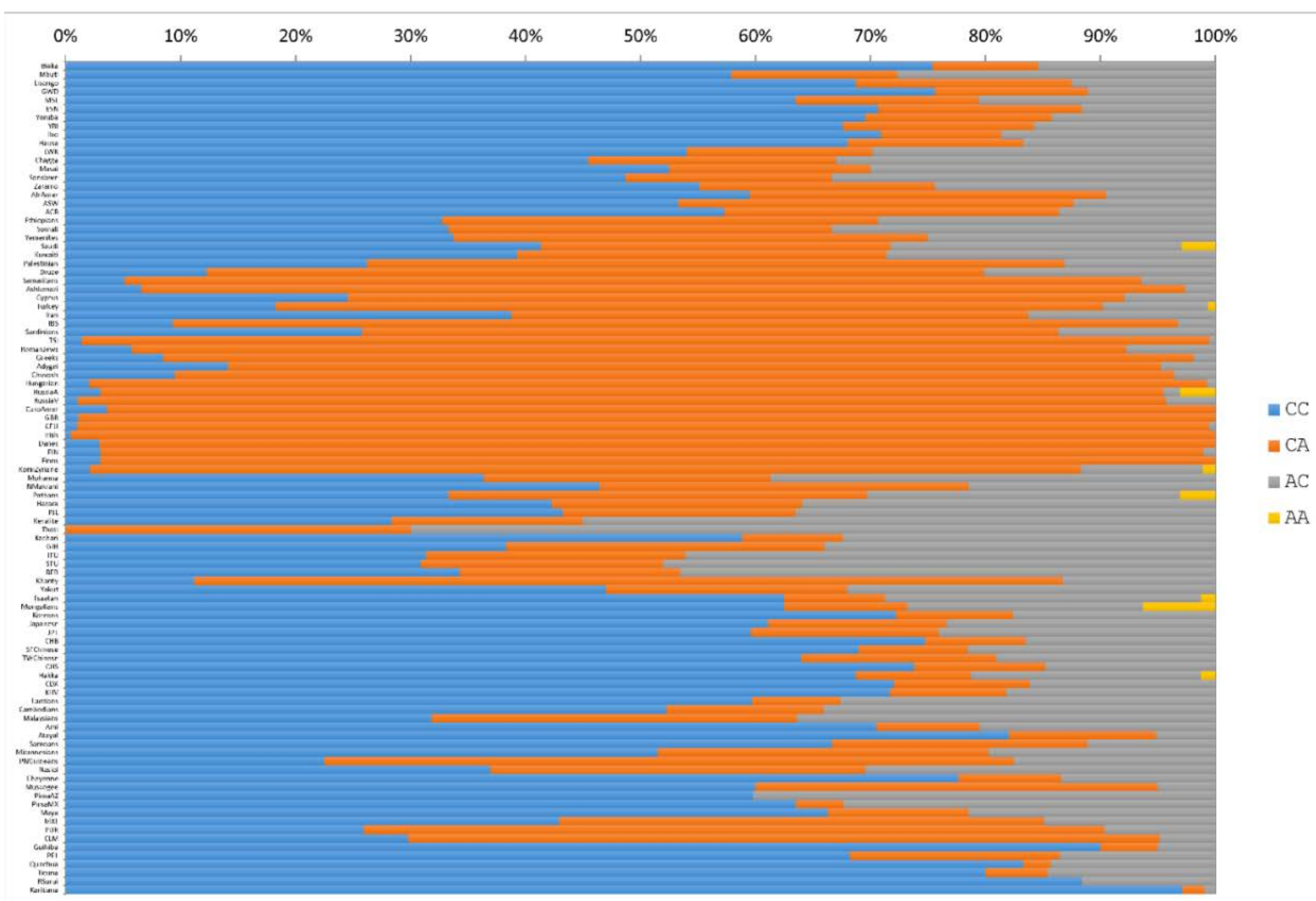

Microhaplotype MH05KK-123

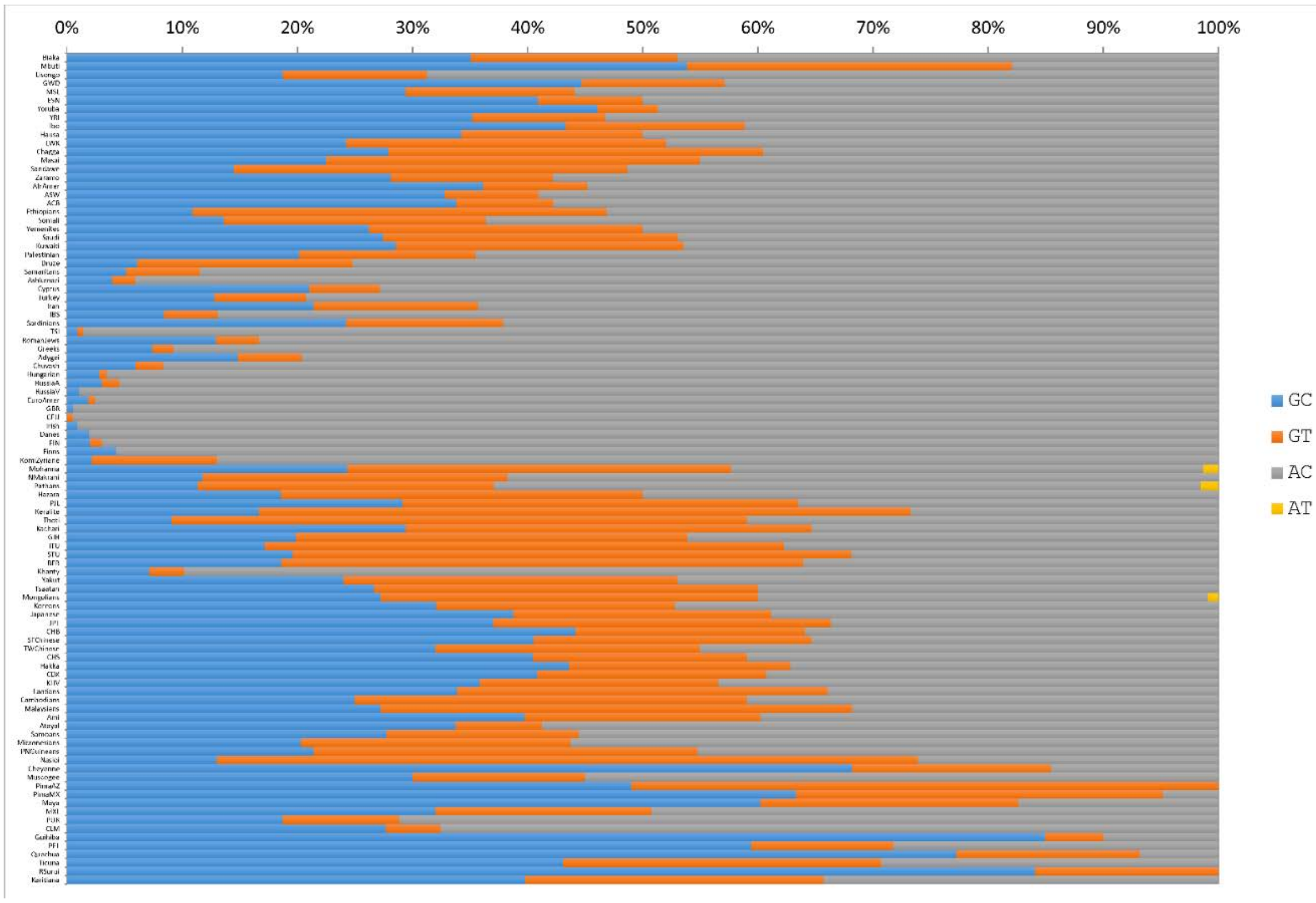

Microhaplotype MH05KK-124

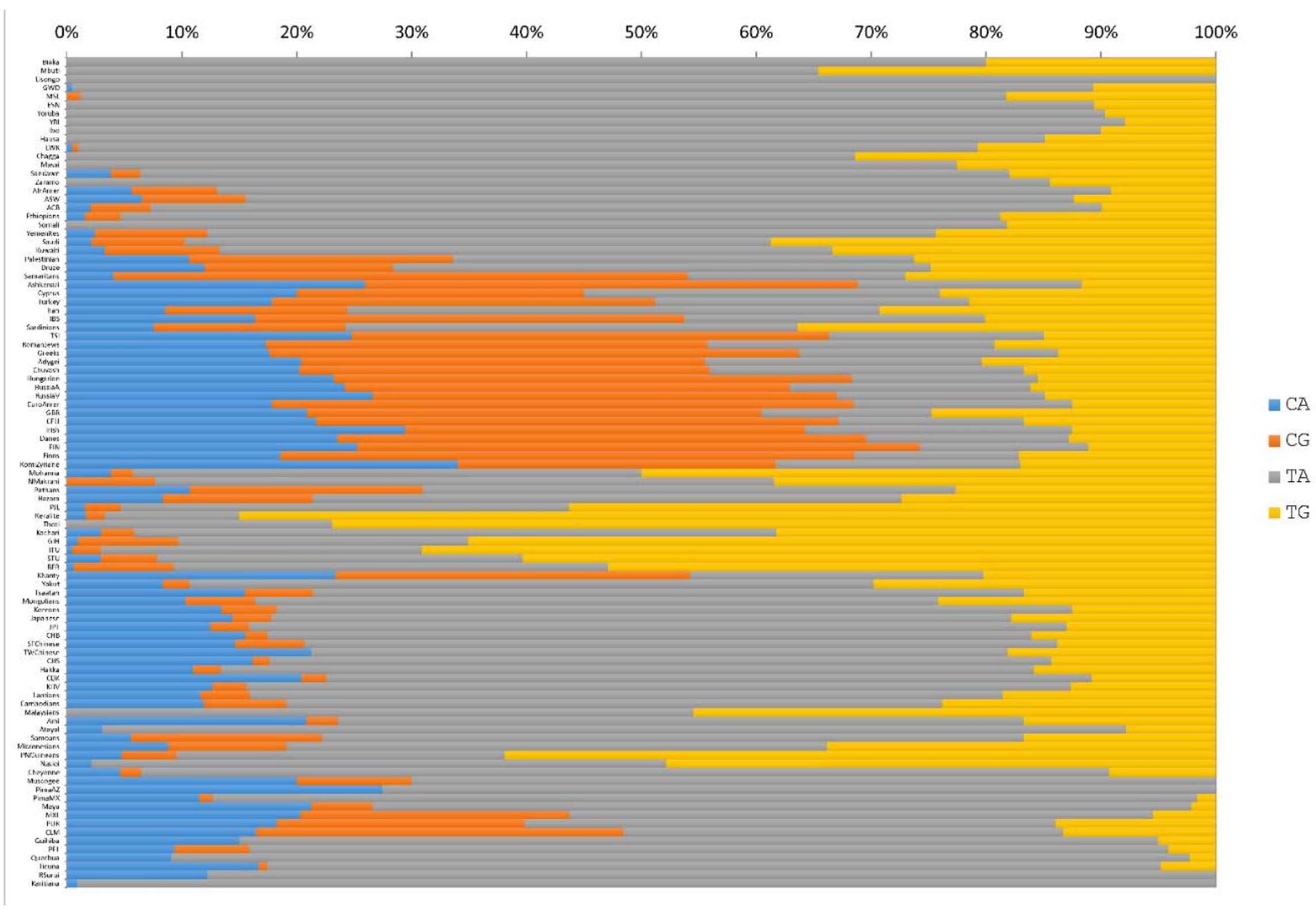

Supplemental Figure S2. Allele frequency bar graphs for the two loci with highest I_n values.

Microhaplotype mh14KK-101

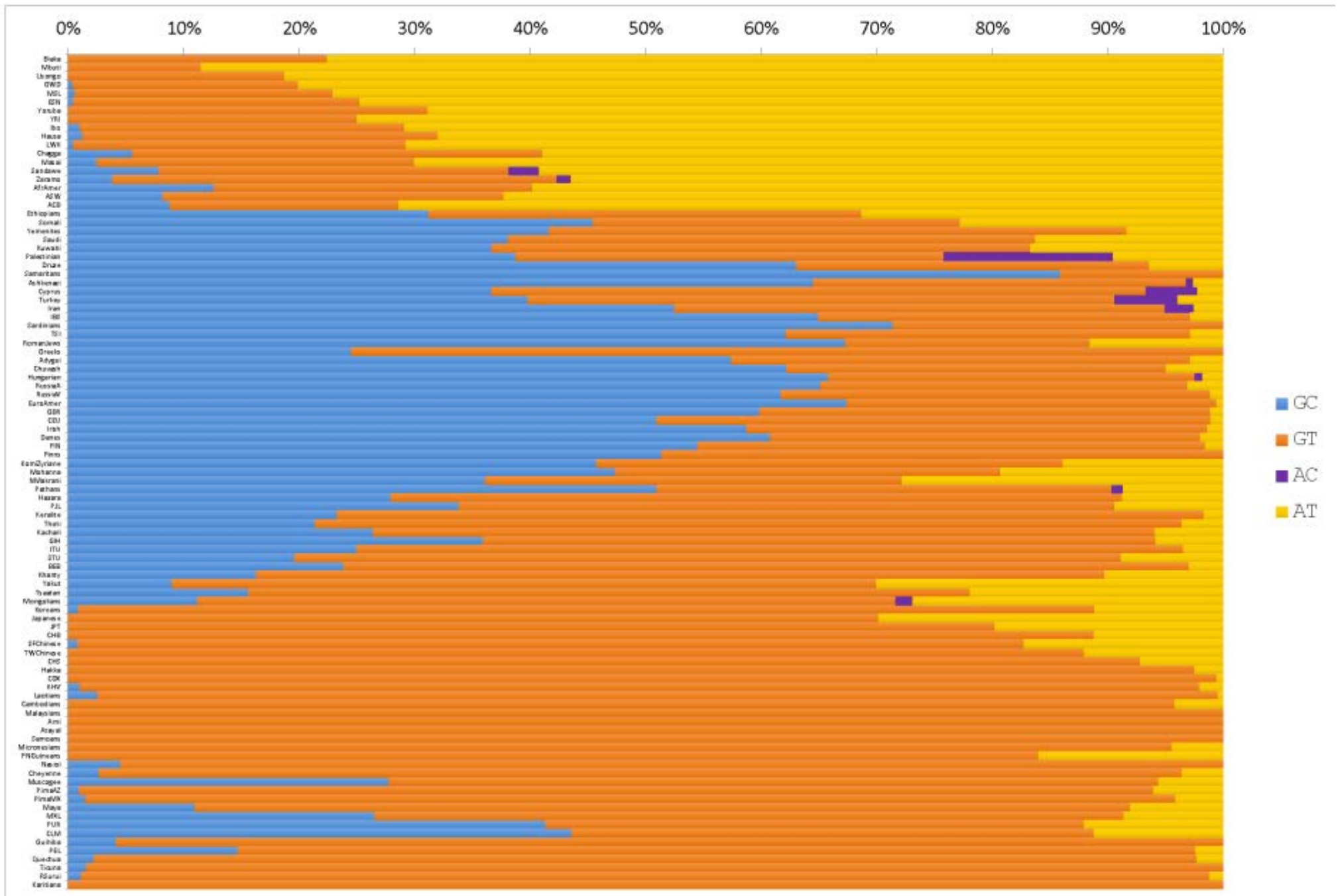

Microhaplotype Mh02KK-003

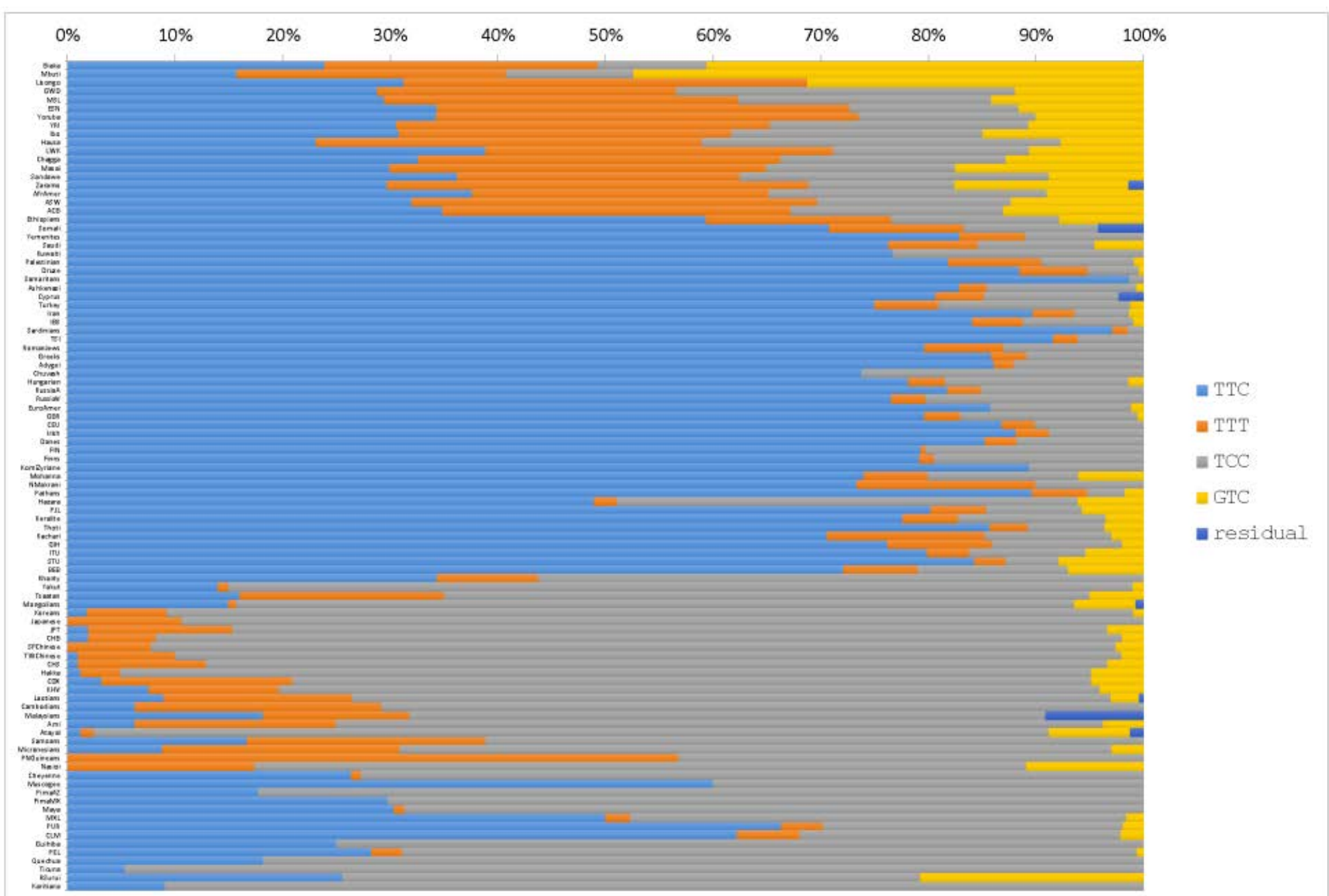

Supplement: Supplementary file 1 [file 414_2017_1748_MOESM1_ESM.pdf]
